# Transcriptomic imputation of genetic risk variants uncovers novel whole-blood biomarkers of Parkinson’s disease

**DOI:** 10.1038/s41531-024-00698-y

**Published:** 2024-05-08

**Authors:** Gabriel Chew, Aaron Shengting Mai, John F. Ouyang, Yueyue Qi, Yinxia Chao, Qing Wang, Enrico Petretto, Eng-King Tan

**Affiliations:** 1 Duke-National University of Singapore Medical School, Singapore, Singapore; 2 Department of Neurology, National Neuroscience Institute, Singapore, Singapore; 3 Yong Loo Lin School of Medicine, National University of Singapore, Singapore, Singapore; 4 Department of Neurology, Singapore General Hospital, Singapore, Singapore; 5 Department of Neurology, Zhujiang Hospital, Southern Medical University, Guangzhou, China

**Keywords:** Parkinson's disease, Genetics

## Abstract

Blood-based gene expression signatures could potentially be used as biomarkers for PD. However, it is unclear whether genetically-regulated transcriptomic signatures can provide novel gene candidates for use as PD biomarkers. We leveraged on the Genotype-Tissue Expression (GTEx) database to impute whole-blood transcriptomic expression using summary statistics of three large-scale PD GWAS. A random forest classifier was used with the consensus whole-blood imputed gene signature (IGS) to discriminate between cases and controls. Outcome measures included Area under the Curve (AUC) of Receiver Operating Characteristic (ROC) Curve. We demonstrated that the IGS (*n* = 37 genes) is conserved across PD GWAS studies and brain tissues. IGS discriminated between cases and controls in an independent whole-blood RNA-sequencing study (1176 PD, 254 prodromal, and 860 healthy controls) with mean AUC and accuracy of 64.8% and 69.4% for PD cohort, and 78.8% and 74% for prodromal cohort. *PATL2* was the top-performing imputed gene in both PD and prodromal PD cohorts, whose classifier performance varied with biological sex (higher performance for males and females in the PD and prodromal PD, respectively). Single-cell RNA-sequencing studies (scRNA-seq) of healthy humans and PD patients found *PATL2* to be enriched in terminal effector CD8+ and cytotoxic CD4+ cells, whose proportions are both increased in PD patients. We demonstrated the utility of GWAS transcriptomic imputation in identifying novel whole-blood transcriptomic signatures which could be leveraged upon for PD biomarker derivation. We identified *PATL2* as a potential biomarker in both clinical and prodromic PD.

## Introduction

Parkinson’s disease (PD) is a prevalent neurodegenerative disease typified by tremors, limb rigidity, akinesia/bradykinesia, and postural instability^1^. Although much progress has been made in understanding PD pathogenesis, PD diagnosis is still primarily based on clinical expert opinion^2^. This not only delays detection of early PD, but also prevents objective monitoring of patient’s response to treatment. Therefore, PD biomarkers provide a quantifiable measure of PD likelihood and/or severity which might be useful for diagnosis, patient stratification, and risk assessment. Primarily, PD biomarker research has been focused on identification from patients’ blood and cerebrospinal fluid which offer a minimally invasive method of disease detection and surveillance^3^. In particular, proteins such as α-synuclein, β-amyloid, and neurofilament light chain, which accumulate in pathological brains, have been earmarked as potential diagnostic biomarkers^3^.

However, despite the strong role of genetics in modulating the risk and phenotypic manifestation of PD^4^, the identification of PD biomarkers based on genetic risks is lacking. In particular, genome-wide association studies (GWAS), which have uncovered several potential risk variants in various pathological contexts ranging from PD motor subtypes^5^ to genetic modifiers of PD^6^, offer a wealth of information for PD biomarker identification. Nonetheless, it is unclear whether these GWAS-identified variants are causal or merely an epiphenomenon in relation to PD pathogenesis. To address this fundamental concern, computational tools have been developed to integrate GWAS data with transcriptomic information (e.g., expression quantitative trait loci^7^ (eQTLs)). Specifically, GWAS transcriptomic imputation utilizes population-level genotype expression databases such as the Genotype-Tissue Expression (GTEx) to identify causal genes from individual GWAS^8^. For example, S-PrediXcan constructs a predictive model based on GTEx genotype-expression information before predicting the association of gene and risk variant from GWAS summary statistics^9^. The imputed tissue-specific transcriptomic expression signature is signed with directionality corresponding to the study design and is effectively equivalent to a transcriptomic expression profile. This expression profile has been applied in various in silico analyses ranging from drug repurposing^10^ to identification of novel causal genes^11^.

In this study, we derived a whole-blood based transcriptomic signature from PD risk variants using transcriptomic imputation via S-PrediXcan^9^. The GWAS being studied include i) a large-scale meta-analysis of 17 PD GWAS consisting of 37,688 cases, 18,618 proxy-cases, and 1.4 million controls^12^, ii) male-specific meta-analysis consisting of 13,020 cases, 7,936 proxy-cases, and 89,660 controls^13^, and iii) female-specific meta-analysis consisting 7947 cases, 5473 proxy-cases, and 90,662 controls^13^. Selection of these GWAS was based on their large sample size (*n* > 100,000) and consideration of multiple individual GWAS and study populations. We subsequently validated the utility of the imputed signature as PD biomarkers in silico by quantifying its performance in classifying cases and healthy controls in male and female-specific PD and prodromal PD cohorts. Single-cell RNA-sequencing (scRNA-seq) studies of whole-blood from PD and healthy individuals were further utilized for uncovering cell type specificities underlying the imputed signature.

## Results

### Transcriptomic Imputation of PD GWAS summary statistics uncovers shared and sex-specific PD risk signatures in whole blood

**Fig. 1:**
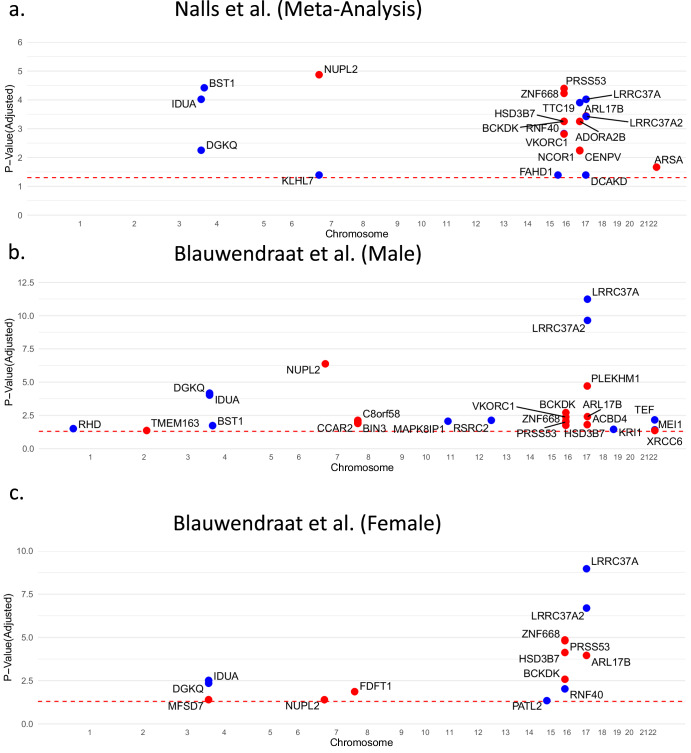
Transcriptomic Imputation of PD GWAS Summary Statistics Uncovers Shared and Sex-specific PD Risk Signatures in Whole Blood. **a** Manhattan plot of imputed transcriptomic expression profile using Genotype-Tissue Expression (GTEx) whole-blood tissue as reference for Blauwendraat et al. (Female) GWAS (7947 cases, 5473 proxy-cases, and 90,662 controls). Significant genes are highlighted in red (upregulated in PD) and blue (downregulated), within-tissue Benjamini-Hochberg adjusted *p* value < 0.05 (dotted red line). **b** Manhattan plot of imputed transcriptomic expression profile using Genotype-Tissue Expression (GTEx) whole-blood tissue as reference for Blauwendraat et al. (Male) GWAS (13,020 cases, 7936 proxy-cases, and 89,660 controls). Significant genes are highlighted in red (upregulated in PD) and blue (downregulated), within-tissue Benjamini-Hochberg adjusted *p* value < 0.05 (dotted red line). **c** Manhattan plot of imputed transcriptomic expression profile using Genotype-Tissue Expression (GTEx) whole-blood tissue as reference for Nalls et al. (Male) GWAS (37,688 cases, 18,618 proxy-cases, and 1.4 million controls). Significant genes are highlighted in red (upregulated in PD) and blue (downregulated), within-tissue Benjamini-Hochberg adjusted *p* value < 0.05 (dotted red line).

Using GTEx (v7), we first applied S-PrediXcan on the largest PD meta-analysis (Nalls et al.) which aggregated data from both female and male study subjects. Using whole-blood as the tissue of reference, 21 significant risk genes were identified (adjusted *p* value < 0.05, Supplementary Data 1) including LRRC37A, LRRC37A2, and DCAKD, which are located near the microtubule-associated protein tau (MAPT) region in which haplotypes associate with the modulation of risk of PD and phenotypic manifestations (Fig. 1a)^14,15^. The risk gene with the strongest signal, NUPL2, was previously identified from a weighted gene coexpression network analysis (WGCNA) of GTEx data and was implicated by an analysis combining genetic and epigenetic level of information^16^. Novel genes and their associated functions were also identified. For example, TTC19, which is essential for the formation of complexes in mitochondrial electron transport chain, is cleaved by the protease PARL which in turn is associated with mitochondrial dysfunction in PD^17,18^. Unsurprisingly, a majority of the identified genes were previously implicated in separate transcriptome-wide association studies (TWAS) (RNF40^19^ and VKORC1^19^) and GWAS meta-analyses (BST1^20^, DGK1^21^, IDUA^21^, and HSD3B7^22^).

Epidemiological studies have also shown that males are 1.5–2 times more likely to develop PD compared to females^23^. Therefore, we hypothesized that transcriptomic imputation of sex-specific GWAS would not only expand the identification of risk genes, but also highlight sex-specific risk genes which would otherwise be masked by the aggregated analysis of both sexes. Therefore, we further employed S-PrediXcan in 2 sex-specific GWAS meta-analyses^13^ and yielded 25 and 14 significant risk genes (adjusted *p* value < 0.05, Supplementary Data 2) for the male and female studies respectively (Fig. 1b, c). We focused on the significant risk genes and assessed i) male/female-specific risk genes and ii) risk genes undetected in the aggregated analysis (Supplementary Fig. 1a, b). We noted four female-specific risk genes—RNF40, PATL2, MFSD7, and FDFT1—with the latter three being undetected in the aggregated GWAS. FDFT1 is involved in the cholesterol biosynthesis pathway^24^, while MFSD7 belongs to the solute carrier family^25^ whose member MFSD2A was found to be an important transporter of omega-3 fatty acid^26^, suggesting the role of lipid metabolism in PD pathogenesis. There were 15 male-specific risk genes with 13 of them not previously detected in the aggregated analysis. Importantly, the most upregulated male-specific risk gene, PLEKHM1, is a key regulator of autophagosome-lysosome fusion^27^, implicating protein accumulation as a hallmark of neurodegenerative diseases. Conversely, the most downregulated male-specific risk gene TEF is associated with the human circadian rhythm and has been associated with increased PD progression^28^, perhaps reflecting REM sleep disturbance which is common in PD^29^. Unsurprisingly, the ten genes significant in both the male and female-specific analyses were all detected in the aggregated analysis (Supplementary Fig. 1A, B), reflecting shared common risk variants across different study populations and biological sex. Of note, the MAPT region-associated genes LRRC37A and LRRC37A2 were the most significant risk genes in both the female and male imputed signatures. Overall, transcriptomic imputation of two additional, sex-specific GWAS meta-analyses identified novel risk genes with respect to the aggregated analyses and revealed sex-specific differences in the imputed signatures. Combining the significant imputed risk genes from all 3 PD meta-analyses allowed derivation of a consensus whole-blood-based imputed gene signature (IGS) consisting of 37 genes (Supplementary Table 1).

### Conservation of whole-blood IGS across GWAS and brain tissues

**Fig. 2:**
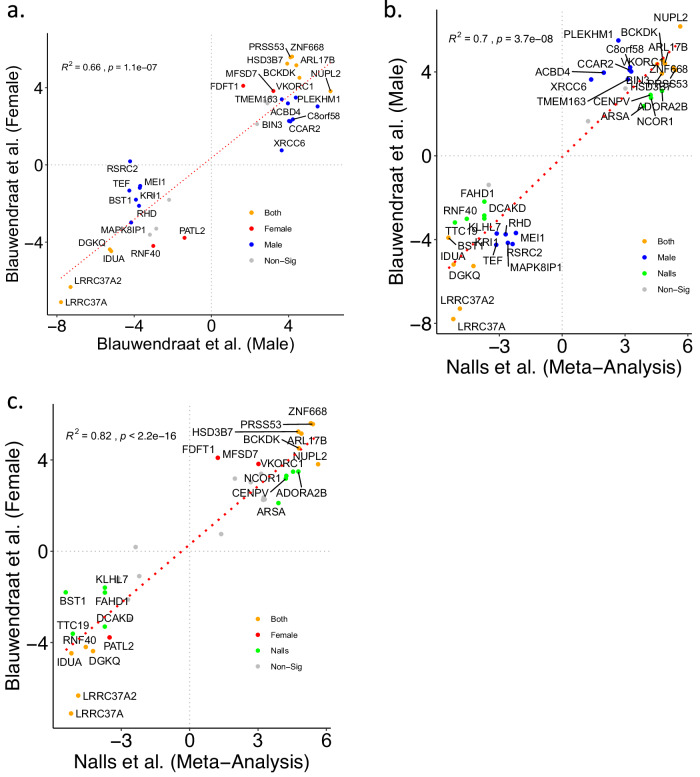
Conservation of whole-blood IGS across GWAS and brain tissues. Scatter plots of z-scores for consensus imputed gene signature (IGS) (*n* = 37 genes) for (**a**) Blauwendraat et al. (Female) (y-axis) and Nalls et al. (x-axis), Spearman correlation, *R*^2^ = 0.82, *p* < 2.2 × 10^−16^. **b** Blauwendraat et al. (Male) (y-axis) and Nalls et al. (x-axis), Spearman correlation, *R*^2^ = 0.7, *p* = 3.7 × 10^−8^. **c** Blauwendraat et al. (Female) (y-axis) and Blauwendraat et al. (Male) (axis), Spearman correlation, *R*^2^ = 0.66, *p* < 1.1 × 10^−7^. Genes are annotated in color to indicate gene set membership - Blauwendraat et al. (Female) (red), Blauwendraat et al. (Male) (blue), Nalls et al. (green), present in both gene signatures in respective pairwise comparison (orange).

Given the differences in demographics, sample sizes, patient variation, clinical and/or pathological criteria utilized between the GWAS, we sought to ascertain whether the IGS (*n* = 37 genes) is conserved across the 3 GWAS. Comparing the consensus IGS z-scores of the male and female GWAS, we observed a strong correlation indicating conservation of the IGS in both the male-specific and female-specific GWAS (Fig. 2a, Spearman correlation, *R*^2^ = 0.66, *p* value = 1.1 × 10^−7^). This is unsurprising given that both GWAS were derived from the same study methodology which had previously found high genetic regulation (*R*_g_ = 0.877) between the female and male GWAS^13^. Out of the 29 imputed genes present in the female and male GWAS, 28 (96.5%) showed concordant directionality of effect. Comparing the sex-specific GWAS to the aggregated GWAS^12^, the IGS is similarly strongly conserved in both of the comparisons with male GWAS (Fig. 2b, Spearman correlation, *R*^2^ = 0.7, *p* value = 3.7 × 10^−8^) and female GWAS (Fig. 2c, Spearman correlation, *R*^2^ = 0.82, *p* value < 2.2 × 10^−16^). Concordance of directionality between the aggregated GWAS and the male-specific/female specific GWAS are 100% and 95.8% (23/24 genes) respectively. The sole discordant gene in all pairwise comparisons, RSRC2, was found to be downregulated in murine 6-OHDA-denervated striatum 1–6 h after L-DOPA administration^30^, potentially reflecting differences in L-DOPA responses in males and females^31^.

Various studies have observed dynamic transcriptomic and cell type proportion changes during PD pathogenesis^32–36^, which could potentially form the basis for transcriptomic biomarkers in PD. However, whole-blood transcriptomic changes might not necessarily reflect the transcriptomic changes in the brain given differences in PD pathogenesis, tissue composition, peripheral and central inflammatory mechanisms^37^, and immune responses to a-synuclein^38^. Therefore, we investigated the extent to which the whole-blood based IGS is conserved in the brain tissue imputed signatures. To accomplish this, we implemented S-PrediXcan on the summary statistics of all three GWAS (Nalls et al., Blauwendraat et al. (male), and Blauwendraat et al. (female)) using all the central nervous system (CNS) tissues in the GTEx (v7) database as references (adjusted *p* value < 0.05). These references included subcortical tissue (e.g., Amygdala and Substantia Nigra.), cortical tissue (e.g., Frontal Cortex (BA9) and Cortex), and cerebellum tissue. For the aggregated GWAS, 6 out of 21 genes (28.6%) were significantly enriched only in whole-blood. These genes included BST1, IDUA, ARL17B, BCKDK, DCAKD, and KLHL7 (Supplementary Fig. 2A). For the remaining 15 genes, only 2 (DGKQ and HSD3B7) were discordant in terms of directionality. For the male-specific GWAS, 10 out of 25 genes (40%) were significantly enriched only in whole-blood, including IDUA, BCKDK, TEF, RSRC2, MAPK8IP1, PRSS53, CCAR2, ACBD4, BST1, and XRCC6 (Supplementary Fig. 2B). 3 out of the shared 15 genes (DGKQ, HSD3B7, and PLEKHM1) were discordant in terms of directionality. For the female-specific GWAS, 7 out of 14 genes (50%) were specific to whole-blood, including PRSS53, ARL17B, BCKDK, IDUA, FDFT1, MFSD7, and PATL2 (Supplementary Fig. 2C), while three out of the remaining seven genes (DGKQ, HSD3B7, and PATL2) were discordant in terms of directionality. Importantly, all three imputed signatures significantly overlapped with CNS-imputed signatures (hypergeometric test, *p* value < 10^−10^) (Supplementary Fig. 3A), suggesting that the whole-blood imputed signatures are present in CNS tissues. Overall, we observed a substantial conservation of whole-blood IGSes in both the three GWAS and CNS tissue.

### In silico validation of whole-blood IGS as biomarkers using a random forest classifier

**Fig. 3:**
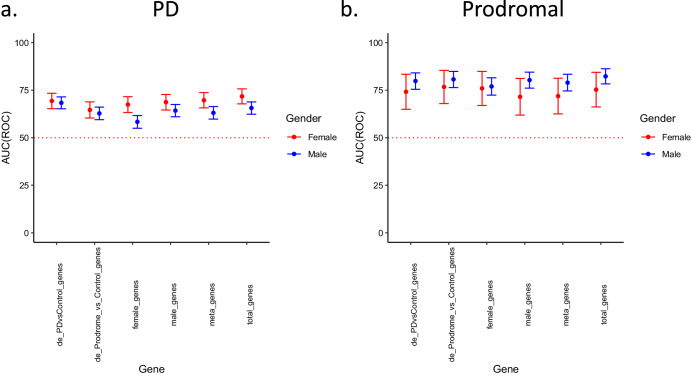
In Silico validation of Whole-Blood IGS as biomarkers using a random forest classifier. Area-under-the-Curve (AUC) of receiver operating curve (ROC) for six gene sets including i) top differentially expressed genes (DEGs) for PD vs Control (*n* = 35), ii) top DEGs for prodromal vs Control (*n* = 35), iii) consensus imputed gene signature (IGS) (*n* = 35), iv) Blauwendraat et al. (male) IGS (*n* = 24), v) Blauwendraat et al. (female) IGS (*n* = 12), vi) Nalls et al. (*n* = 20) for (**a**) PD cohorts in both males (550/550 cases/controls) and females (319/319 cases/controls), (**b**) prodromal cohorts in both males (198/198 cases/controls) and females (56/56 cases/controls). 95% confidence intervals were derived from 2000 stratified bootstrap replicates. Error bars reflect the 95% confidence intervals of the corresponding estimate.

Given that the whole-blood IGS represents a consensus of genetically regulated transcriptomic signature based on PD risk variant information, we sought to quantify its utility as PD blood biomarkers. To achieve this, we leveraged on a recently published whole-blood, bulk RNA-sequencing meta-analysis consisting of 1716 PD (1119 male and 597 female), 254 prodromal (198 male and 56 female), and 869 (550 male and 319 female) healthy controls. Individuals classified with prodromal PD were characterized by the presence of REM sleep behavior disorder, hyposmia, positive dopamine transporter (DAT) SPECT scans, and evidence of genetic risk and positive family history^39^. Using a random forest classifier (See Methods), we evaluated the performance of the GWAS-specific and consensus whole-blood IGS by quantifying the accuracy, area under the curve for the receiver operating curve (AUC-ROC), and area under the curve for the precision-recall curve (AUC-PR) (Supplementary Table 2). As a positive control, we also evaluated the performance of an equivalent set of differentially expressed genes (DEGs) in PD and prodromal PD (Fig. 3a, b). Firstly, the whole-blood IGSes discriminated cases from controls better for prodromal PD than for PD with average AUC-ROC of 79.6% and 73.7% in males and females respectively in prodromal PD compared to corresponding AUC-ROC of 62.8% and 69.4% in PD. Importantly, the consensus IGS did not significantly overlap with DEGs in both PD (hypergeometric test, *p* value = 0.997) and prodromal PD (hypergeometric test, *p* value = 0.339) cohorts. Therefore, the better performance in prodromal PD is not related to a greater proportion of the consensus IGS (*n* = 35) being differentially expressed in prodromal PD (57.1%, 20/35) than in PD (20%, 7/35). This, coupled with the observation that log fold changes from bulk RNA-seq dataset and imputed z-scores from IGS did not show significant association in both PD (Supplementary Fig. 4A, Spearman correlation, *R*^2^ = 0.017, *p* value = 0.46) and prodromal PD cohorts (Supplementary Fig. 4B, Spearman correlation, *R*^2^ = 0.092, *p* value = 0.082), suggests that the consensus IGS is not conserved in the bulk RNA-seq datasets. Altogether, these observations could be due to the derivation of the imputed gene from reference transcriptomic dataset (GTEx) and SNP-level information which is in turn based on large samples of healthy populations, while the DEG is derived from actual quantification of mRNA levels in pathological and healthy whole-blood samples. Secondly, for the PD cohort, the IGSes (female-specific, male-specific, aggregated (Nalls et al), and consensus) have differential performance in females and males with higher performance in the former compared to the latter. This observation was reversed in the prodromal cohort albeit to a smaller extent. Interestingly, the DEGs (PD and Prodromal) did not exhibit this differential performance in males and females. Thirdly, aside from the female PD cohort, the sex-specific whole-blood IGSes (male, *n* = 25 genes; female, *n* = 14 genes) performed better within cohorts of their corresponding biological sex, reflecting the sex-specific differences in PD/prodromal PD pathogenesis. Supporting this observation, the aggregated IGS (Nalls et al., *n* = 21 genes) performed marginally worse than the sex-specific IGSes except in the female PD cohort. Fourthly, the consensus IGS (*n* = 37 genes) outperformed all other IGSes except in the female prodromal PD cohort. The consensus IGS also performed similarly to an equivalent set of DEGs with higher accuracy and AUC for the female PD and male Prodromal cohorts. Overall, we demonstrated that risk variant information (GWAS summary statistics) can be leveraged upon to infer blood-based biomarkers through transcriptomic imputation, with a priori IGS performing equivalently to a posteriori DEGs. In addition, we also validated the specificity of the female-specific and male-specific IGSes in an independent whole-blood PD/prodromal PD transcriptomic dataset.

### PATL2 is the top classifier imputed gene in both PD and Prodromal PD Cohorts

**Fig. 4:**
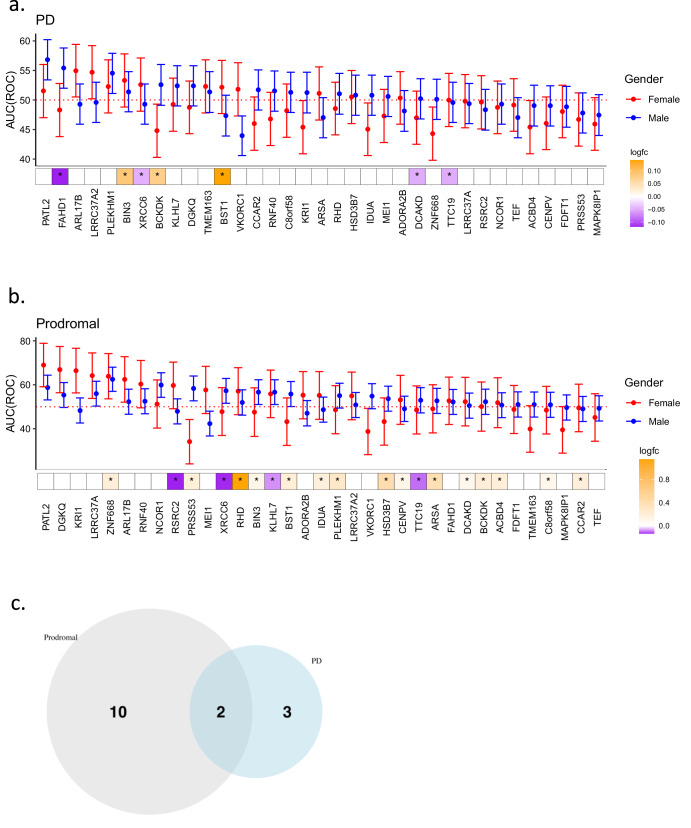
PATL2 is the top classifier imputed gene in both PD and Prodromal PD cohorts. Single-gene area-under-the-curve (AUC) of receiver operating curve (ROC) and status as differential expressed gene (with corresponding log fold changes) for consensus IGS (*n* = 35) for (**a**) PD cohorts in both males (550/550 cases/controls) and females (319/319 cases/controls), (**b**) prodromal cohorts in both males (198/198 cases/controls) and females (56/56 cases/controls) 95% confidence intervals were derived from 2000 stratified bootstrap replicates. **c** Venn diagram of significant imputed risk genes (defined as single-gene AUC(ROC) > 50% in either male or female) in PD and prodromal cohorts. Error bars reflect the 95% confidence intervals of the corresponding estimate.

In order to ascertain which gene in the consensus IGS has the highest performance as a solitary biomarker, we employed the same random forest classifier protocol for each imputed gene in males and females for both PD and prodromal cohorts (Fig. 4a, b). Similar to the observations made in the previous analysis, AUC-ROC were higher in prodromal cohorts relative to PD cohorts with 21.4% (15/70—number of genes with lower tail of AUC-ROC greater than 50%/total number of genes; 12 unique genes) and 7% (5/70, 5 unique genes) of the IGS outperforming random assignment (defined as lower tail of AUC(ROC) higher than 50%) in the prodromal and PD cohorts respectively (Fig. 4c, see *Methods*). Importantly, there is no correspondence between AUC-ROC and whether the gene is differentially expressed (Fig. 4a, b). Comparing these 2 sets of imputed genes, ARL17B and PATL2 were consistently top performers in both PD and prodromal PD cohorts (Fig. 4c). ARL17B, which was identified in the imputation of all 3 GWAS (Supplementary Fig. 1A), is involved in protein trafficking whose level of methylation was found to correlate with SNPs associated with the Parkinson-plus syndrome, progressive supranuclear palsy (PSP)^40^. Importantly, PATL2 has the highest AUC-ROC in both PD and prodromal cohorts. In detail, PATL2 has a mean AUC-ROC of 52 and 56% for females and males in the PD cohort, and 69 and 59% in females and males in the prodromal cohort. Therefore, the classifier performance of PATL2 is dependent on the biological sex with higher performance for males and females in the PD and prodromal PD cohorts respectively, suggesting differential effect of PATL2 in both sexes for PD and prodromal PD pathogenesis. Supporting this observation, PATL2 was also only identified in the imputation of female-specific PD GWAS (Fig. 1c). To further disentangle the cell type specificities of the consensus IGS, we leveraged on a scRNA-seq study of 7551 blood cells isolated from healthy human subjects (Supplementary Fig. 5A)^41^. Importantly, PATL2 is significantly enriched in T-lymphocytes and B-Lymphocytes (Supplementary Fig. 5A). Further analysis of a T-lymphocyte-specific scRNA-seq study of blood isolated from eight PD patients and six healthy controls revealed that PATL2 is significantly enriched in terminal effector CD8+ and cytotoxic CD4 + T-lymphocytes (Supplementary Fig. 5B) whose populations were both found to be significantly expanded in PD^42^. These findings demonstrate that imputed risk genes could be potential single-gene biomarkers with PATL2 observed as the top performing classifier gene with specific enrichment in pathological T-lymphocyte subsets in PD.

## Discussion

In this study, we obtained novel PD transcriptomic signatures by employing transcriptomic imputation of three PD GWAS summary statistics (one large-scale, aggregated meta-analysis, one female-specific, and one male-specific). In doing so, we disentangled sex-specific risk genes and derived a consensus imputed gene signature (IGS) in whole blood. Although the integration of GWAS information with gene expression data has already been employed in the identification of causal PD risk genes, these studies predominantly focused on the CNS^16,19,43^. Notably, Yang et al. identified 44 and 29 PD risk genes in dorsolateral prefrontal cortex (DLPFC) and monocytes via a TWAS by leveraging on DLPFC bulk transcriptomic dataset from the CommonMind Consortium (CMC)^44^ and 3 independent bulk transcriptomic datasets of peripheral-blood monocytes^43^. In another TWAS, Yao et al. constructed models based on CNS transcriptomic expression, epigenetic annotations, and 4 PD GWAS summary statistics totaling 33,674 cases and 449,056 controls^19^. Similar to our study, Yao et al. observed LRRC37A2 as a key gene driving GWAS signal at its loci. Lastly, Kia et al. integrated PD GWAS with both expression data and methylation data to identify PD disease genes including NUPL2, which demonstrated the strongest signal in our imputed signature of the aggregated GWAS (Nalls et al.)^16^. Comparing our consensus whole-blood IGS (*n* = 37) with signatures generated from these independent transcriptomic association studies, only 13 out of 37 genes were shared, possibly reflecting the differences in genetically regulated gene expression between CNS tissue and whole blood. These differences in causal genes identified could also stem from the fact that both Yao et al. and Kia et al. utilized additional layers of -omic information (i.e., epigenetics) in their integration workflow as well as different PD GWAS interrogated, methodologies, and reference datasets utilized, reflecting the genetic heterogeneity across study populations/demographics in PD.

Regardless, our study focused on whole-blood instead of the CNS because of the greater accessibility of whole-blood as disease biomarkers^45,46^. Significantly, our study also demonstrates a *proof-of-concept* approach in validating the utility of genetically regulated gene expression as PD biomarkers. This validation was performed in the largest whole-blood RNA-seq dataset of PD/prodromal PD to our knowledge^47^. In particular, we showed that the imputed IGSes have differential discriminating performance for i) PD vs prodromal cohorts and ii) male vs female cohorts, while performing equivalently to a posteriori set of DEGs (PD/prodromal vs controls) derived from the same whole-blood RNA-seq dataset. The higher classifier performance in prodromal cohorts relative to PD cohorts was unexpected given that the three GWASes were based on PD as the clinical trait of study^12,13^. A possible explanation could lie in the differences in neutrophil/lymphocyte ratio between PD and prodromal patients. Craig et al. recently showed that, while the whole-blood neutrophil/lymphocyte ratio is increased in both PD and prodromal patients relative to healthy subjects, the rate of change of neutrophil marker expression increases in prodromal patients but remains unchanged in PD^47^. With respect to the IGSes’ differential performance in male and female cohorts, we observed higher classifier performance for females in the PD cohort and for males in the prodromal cohort, which might reflect the role of biological sex in PD pathogenesis. Expectedly, the DEGs (which had accounted for biological sex as a covariate) did not show this differential performance between males and females, supporting the IGSes’ sex-specific performance. Lastly, other factors such as differences in population size (PD cohort » prodromal cohort) and demographics could also play a role in explaining these observations.

Extending the utility of IGS as potential PD whole-blood biomarkers, we uncovered *PATL2* as the top risk gene in terms of classifier performance and found its enrichment in pathological T lymphocyte subsets (cytotoxic CD4^+^ and terminal effector CD8^+^ subsets) which are both expanded in PD pathogenesis. Specifically, Wang et al. found that terminal effector CD8^+^ T-lymphocytes originated from central memory CD8^+^ T-lymphocytes and are enriched for cell adhesion, while cytotoxic CD4^+^ T-lymphocytes represent an infiltrating subset in PD pathogenesis^42^. Regarding the role of T-lymphocytes in PD pathogenesis, their infiltration has been speculated to initiate cytotoxic attack and neuronal death, preceding the process of a-synuclein aggregation^48^. Other studies, however, have suggested that a-synuclein acts as an antigenic epitome that drives CD8+ and CD4 + T-lymphocyte responses, thereby causing dopaminergic neurodegeneration^49^. Currently, there is no consensus to the sequence of events, but T-lymphocyte infiltration appears to at least occur concurrently with a-synuclein accumulation and neuronal death in PD^48,49^. However, despite being enriched in the expanded T-lymphocyte population as seen in PD, PATL2 expression and its enrichment in infiltrating T-lymphocytes remain unaffected by treatment status. Overall, our results suggest PATL2 as a potential peripheral whole-blood biomarker and contributor to PD pathogenesis by potentially regulating T-lymphocyte infiltration.

Leveraging on the recent Human Protein Atlas, we observed specific PATL2 enrichment in immune cells of lymphoid lineage including Natural-Killer (NK) cells, T-lymphocytes, B-lymphocytes, dendritic cells, and plasma cells (https://www.proteinatlas.org/)^50^. This might be important given that various independent studies, including Wang et al., have found significant changes in proportions of different immune cellular subpopulations including NK cells^51^, gamma delta T-lymphocytes^51^, and follicular T-lymphocytes^52^ in PD pathogenesis. This raises the intriguing role of peripheral inflammation as a key player in PD pathogenesis. Indeed, a key mechanism of the interplay between peripheral and central pathological processes lies in how peripheral inflammation polarizes microglia into a pro-inflammatory phenotype in neurodegenerative diseases such as PD^53^, Lewy Body Dementia^54^, and Alzheimer’s Disease^55^. With respect to T-lymphocytes, CD4^+^ T-lymphocytes have been shown to modulate neuroinflammation in a mouse model of PD^56^, while a-synuclein can also induce T-lymphocyte response in PD^57^.

Our study found the classifier performance of PATL2 to be notably better in prodromal PD as compared with PD. Additionally, PATL2 performed better for females in the prodromal cohort, whereas the converse is true for the PD cohort. This finding may point towards a sex-specific involvement of the PATL2 gene in PD pathogenesis, such as in PD patients with versus without a prodromal phase. The datasets we used also found PATL2 expression to be downregulated only in females with PD (Supplementary Fig. 2), which stands in contrast with prior literature that demonstrated an age-related upregulation of PATL2 expression in human lymphocytes^58^. Thus, the downregulation of PATL2 may be involved in the underlying pathogenetic process of both PD and prodromal PD, especially in female patients. Further supporting this point is the finding of PATL2’s specific role in oocyte maturation with PATL2 deficiency contributing to oocyte meiotic deficiency (OMD)^59,60^. Given that prodromal PD is hallmarked by nonmotor symptoms, and that many of the nonmotor symptoms (e.g., olfactory dysfunction, constipation, and depression) in the prodromal PD diagnostic criteria were more prevalent in women than men^61,62^, we additionally hypothesize that PATL2 is also involved in the mechanisms underlying the development of nonmotor symptoms. The difference in the classifier performance of PATL2 may therefore be a result of the different demographics and clinical presentations of PD versus prodromal PD.

PATL2, however, remains a poorly studied gene especially within the neurodegenerative diseases. Various meta-analyses on human transcriptome studying aging, Alzheimer’s disease, as well as other neurodegenerative conditions (e.g., Huntington’s disease, progressive supranuclear palsy, amyotrophic lateral sclerosis), have not found a significant association between PATL2 expression levels and disease risk^63–65^. The role of PATL2 in immune cells remains unclear, other than being upregulated in lymphocytes with increasing age. Further studies are needed to determine if PATL2 has any involvement with other neurodegenerative conditions, especially those that involve synucleinopathies. Moreover, the role of PATL2 in immune cells, especially T-lymphocytes, needs to be elucidated. The findings of our study suggest the potential role of neuroinflammation mediated by peripheral T-lymphocytes, but this should be confirmed by additional transcriptomic and even multi-omics studies, as well as cell cultures and animal models.

In summary, these observations point to peripheral whole-blood T-lymphocytes as key actors of PD pathogenesis whose activity, markers, and/or proportions could be utilized as PD biomarkers. Indeed, a previous transcriptomic meta-analysis of PD and major depressive disorder (MDD), which is often found in patients with prodromal PD and 3–6 years before development of PD motor symptoms^66^, also highlighted PATL2 as a potential whole blood biomarker^67^. However, it should be noted that MDD is not uncommon in the general population and its symptoms alone may not be specific enough to suspect prodromal PD. Unlike previous studies, our study shows that common risk variants (contained within GWAS summary statistics) can be used to predict transcriptomic expression profiles for the derivation of whole blood biomarkers of PD. This approach is orthogonal to transcriptomic derivation of blood-based biomarkers^34,67^. In addition, the imputed signatures reflect aggregated genetic contributions from common risk variants in PD from three large-scale GWAS meta-analyses, and we further validated the signatures in independent transcriptomic datasets across different study populations and disease stages (prodromal and PD).

Nonetheless, there are important limitations to our approach. First, we observed that the imputed signature is not conserved in the validation transcriptomic dataset, which could be attributed to the fact that the transcriptomic imputation step relied on healthy tissue as reference transcriptome dataset (i.e., GTEx). Therefore, given that this reference dataset is used to calculate gene variance and covariance^9^, imputed z-scores might not accurately reflect the direction of effect in disease. Second, the GWAS summary statistics utilized were based mostly on western populations, which limits its applicability in eastern populations given their different genetic risk profiles^68^. Third, imputed signatures reflect a mixture of cellular types, which might limit direct comparison of the imputed signature with transcriptomic profiles derived from bulk-tissue RNA-seq if the proportions of cell types are not comparable. This is especially so with studies showing that bulk transcriptomic differences are largely driven by cell type proportion changes^69^. Fourth, although the IGSes performed well in discriminating cases and controls, the consensus signature (*n* = 37 genes) did not significantly outperform *a posteriori* DEGs. Fifth, our approach for utilizing imputed whole-blood signatures as biomarkers might not be applicable in cases where peripheral blood either does not harbor measurable pathological processes or does not reflect tissue pathogenesis in a clinical relevant manner^70^. Sixth, there are limitations inherent to the GWAS methodology, such as missing heritability (though no current methods can determine all genetic determinants of complex traits). Seventh, our findings are derived from large GWAS involving only subjects of European ancestry and may hence have limited generalizability to other populations, such as Asian and African populations. Lastly, this paper did not compare the classifier performance of PATL2 in PD versus other neurodegenerative conditions, which would provide useful insight into the specificity of PATL2 as a genetic biomarker. This would be an important area for future investigation.

In conclusion, our study demonstrates the derivation of PD whole-blood biomarkers by utilizing genetic risk variant information. We identified PATL2 as a novel genetically regulated risk gene enriched in pathological T-lymphocyte subsets that could be a transcriptomic PD biomarker. Future work could focus on GWAS transcriptomic imputation based on diseased tissue instead of healthy tissues, incorporation of scRNA-seq in deconvoluting bulk signatures, and consideration of more heterogeneous PD GWASes including Asian and African populations.

## Methods

### Transcriptome imputation of genome-wide association studies (GWAS)

Summary statistics were downloaded for aggregated meta-analysis (Nalls et al.), female-specific meta-analysis (Blauwendraat et al.), and male-specific meta-analysis (Blauwendraat et al.) from https://pdgenetics.org/resources which is managed by the International Parkinson’s Disease Genomics Consortium (IPDGC). For all GWAS, ANNOVAR was employed to annotate the variants with the appropriate SNP identifier using reference dataset of hg19 build and dbSNP version 147. Transcriptomic imputation of the summary statistics was performed using S-PrediXcan^11^(https://github.com/hakyimlab/MetaXcan). Briefly, S-PrediXcan utilizes GWAS summary statistics to infer gene-level statistics by utilizing variances and covariances calculated from the reference dataset (in our case, the 1000G reference). S-PrediXcan leverages on pre-calculated weights derived from training dataset. Specifically, we utilized pre-calculated weights and variances with respect to the GTEx v7 Expression Model^9,71,72^. We applied S-PrediXcan for all 48 tissues of reference using covariances and transcriptome prediction model databases from the GTEx v7 pre-calculated references based on European populations (https://predictdb.org/post/2017/11/29/gtex-v7-expression-models/). Next, in order to account for multiple testing, we performed within-tissue p-value adjustment using the Benjamini-Hochberg method which was similarly employed in a transcriptomic imputation study of post-traumatic stress disorder^11^. biomaRt (v2.46.3) (accessed on 4th Jan 2022) was used to identify protein-coding genes and non-protein-coding genes were filtered out for downstream analysis. For plotting of Manhattan plots of whole-blood imputed transcriptome, code was adapted from (https://danielroelfs.com/blog/how-i-create-manhattan-plots-using-ggplot/). Significant genes were defined using threshold of adjusted *p* value lesser than 0.05. Only autosomes were considered. For visualization purposes, non-significant data were downsampled.

### Preprocessing bulk transcriptomic dataset for in silico validation

In silico validation was performed on a bulk transcriptomic RNA-seq dataset consisting of 1716 PD (1119 male and 597 female), 254 prodromal (198 male and 56 female), and 869 (550 male and 319 female) healthy controls from the Parkinson’s Progressive Marker Initiative (PPMI) cohort via LONI IDA (https://ida.loni.usc.edu/)^47^. Prodromal PD cohort is defined as participants that have rapid eye movement (REM) behavior or hyposmia and a DaTscan that shows evidence of dopaminergic deficits^47^. Each of the 4 bulk transcriptomic dataset (PD vs Control (Male), PD vs Control (Female), Prodromal vs Control (Male), and Prodromal vs Control (Female)) was processed as follows, 1) filtering of lowly expressed genes i.e genes without at least a count of 25 in at least 25% of samples with the exception for “PD vs Control (Female)” in which the threshold was set at 20%. 2) Normalization was performed using variance stabilization transformation via DESeq2 (v1.30.1) after considering “Plate” as a potential confounding factor. Prodromal and PD differentially expressed genes (DEGs) were obtained from respective gene lists^47^.

### In silico validation of whole-blood consensus imputed gene signature (IGS)

For each run of the in silico validation pipeline, the number of samples was downsampled in order to balance the number of cases and controls. This was performed randomly using ovun.sample() via ROSE(v0.0.4) with seed set at 42. Random forest classifier was employed using *randomForest()*(v4.6.14) with the number of trees set at 500 and “mtry” (i.e number of variables randomly considered at each split) set as square-root of the number of samples. Performance metrics (“Accuracy”, “AUC (Precision-Recall)”, “AUC (ROC)”) were calculated using performance() via ROCR(v1.0.11). Confidence interval for “AUC(ROC)” was quantified using roc() via pROC (v1.18.0) which computes the 95% confidence interval with 2000 stratified bootstrap replicates. For in silico validation of individual imputed genes, we utilized the same pipeline illustrated above with the exception that “mtry” was set at 1.

### Single-cell RNA-sequencing (scRNA-seq) analysis

For scRNA-seq analysis of healthy human whole-blood, we downloaded scRNA-seq data of 7643 cells which comprised of 32 different cell types and derived from 21 healthy human samples from Gene Expression Omnibus (GEO) (GSE149938)^41^. Seurat (v4.1.0) was used to process the data using the log normalization method with scale factor of 10,000. Highly variable genes were derived using *FindVariableFeatures()* which was set at 5000. RunPCA() and RunUMAP() were employed using 50 dimensions. Cell types were annotated based on the author’s classification including hematopoietic stem cells (HSPCs), B lymphocytes, Natural Killer (NK) cells, T lymphocytes, monocytes, and neutrophils. Cell type markers were calculated using FindAllMarkers() with both minimum log fold change threshold and minimum percentage of cells set at 0.

For scRNA-seq analysis of human T-lymphocytes in healthy individuals and PD patients, we downloaded data from https://zenodo.org/record/3993994#.YgNwVe5Bw1I, which sequenced T-cell enriched blood samples from 8 PD patients and 13 patients^42^. For our analysis, we only considered samples with available annotation for biological sex namely 8 PD patients (P1, P2, P3, P4, P5, P6, P7, and P8) and 6 healthy controls (N1, N2, N3, N4, N5 and N6). Seurat was used to process the data with the same pipeline employed as highlighted above. Cell type markers were derived using FindAllMarkers() with both minimum log fold change threshold and minimum percentage of cells set at 0.25.

### Power calculation

Our power calculation employed the unbalanced one-way analysis of variance (ANOVA) method, which is also the method employed by the GTEx consortium^73^. We adopted the same parameters as the GTEx project since we are similarly examining tissue eQTLs. The expression data was modeled as a lognormal distribution with a log standard deviation of 0.13 within each genotype (i.e., wildtype homozygotes, heterozygotes, mutation homozygotes). The between-genotype difference is set at 0.13, which is a log expression change that is equivalent to the standard deviation within each genotype. We adopted the power calculation formula as suggested by O’Brien and Muller in 1993^74^, which involved the unbalanced one-way ANOVA method to test if a SNP is associated with a gene expression level across the three possible genotypes. To perform the power analysis, we utilized the *powerEQTL* package in R developed by ref. ^75^ As such, to achieve a power of 80%, a minimum of 2189 participants is needed.

### Reporting summary

Further information on research design is available in the Nature Research Reporting Summary linked to this article.

### Supplementary information

Supplemental Information

Reporting Summary

Data Set 1

Data Set 2A (female)

Data Set 2B (male)

## Data Availability

All data analyzed are found in already published studies, and no new original data is generated or analyzed in this study. The datasets used for transcriptomic imputation (namely the meta-analysis by Nalls et al. as well as the male- and female-specific datasets by Blauwendraat et al.) can be accessed at and downloaded directly from the following resource: https://pdgenetics.org/resources.

## Code availability

The code and functions used to perform all statistical analyses in this paper will be available upon request. Additionally, the links leading to the documentation for the key functions employed are provided in the *Methods* section.
