# Supplementary material for: Transcriptomic imputation of genetic risk variants uncovers novel whole-blood biomarkers of Parkinson’s disease
Source: NPJ Parkinsons Dis. 2024 May 8;10:99. doi: 10.1038/s41531-024-00698-y (PMC11078960; doi:10.1038/s41531-024-00698-y)
Supplement: Supplementary file 1 — Supplemental Information [file 41531_2024_698_MOESM1_ESM.pdf]

Contents

Supplementary Figure 1 2
Supplementary Figure 2 3
Supplementary Figure 3 4
Supplementary Figure 4 5
Supplementary Figure 5 6
Supplementary Table 1 7
Supplementary Table 2 8

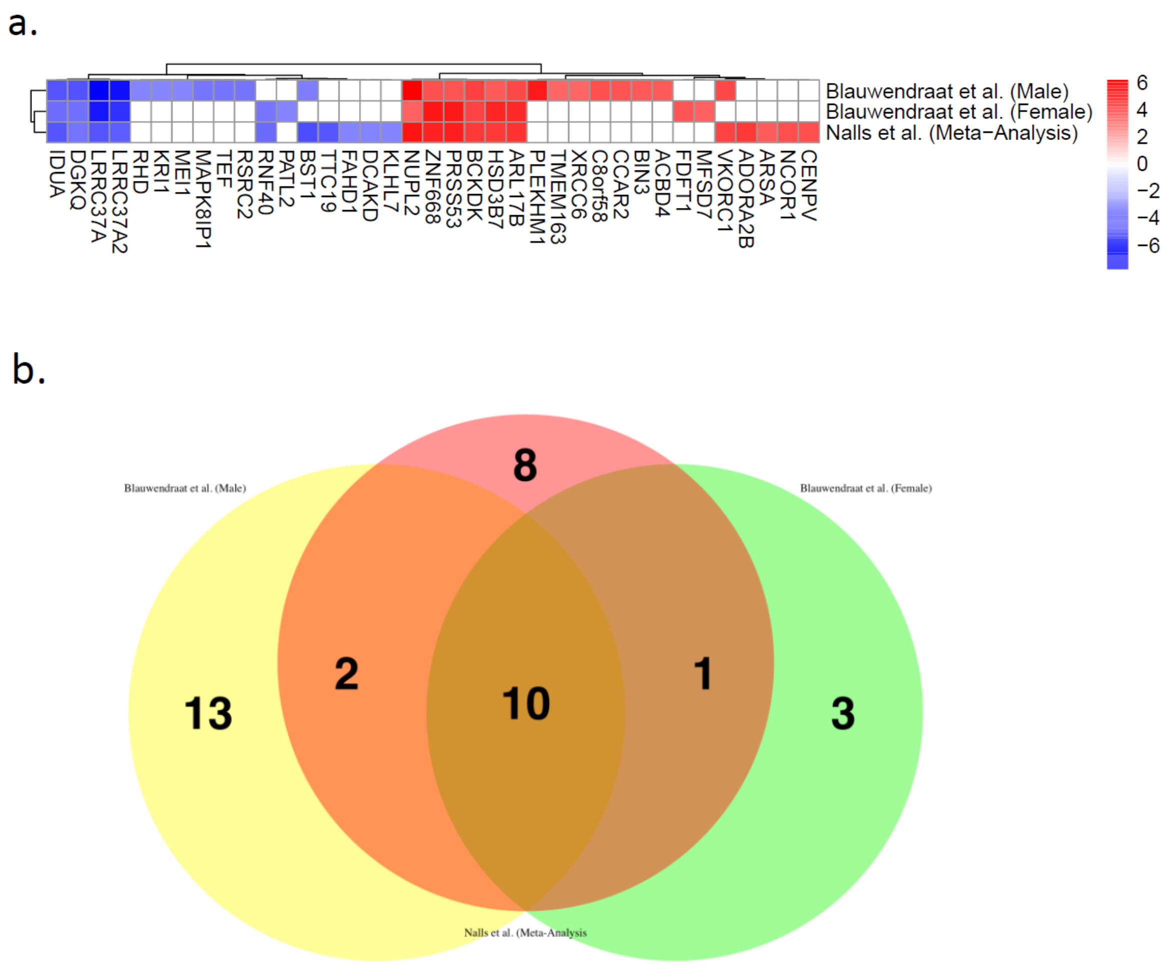

Supplementary Figure 1

a) Heatmap of imputed z-scores for Blauwendraat et al. (Female, 14 genes), Blauwendraat et al. (Male, 25 genes), and Nalls et al. (21 genes). **b)** Venn diagram of Blauwendraat et al. (Female, 14 genes), Blauwendraat et al. (Male, 25 genes), and Nalls et al. (21 genes)

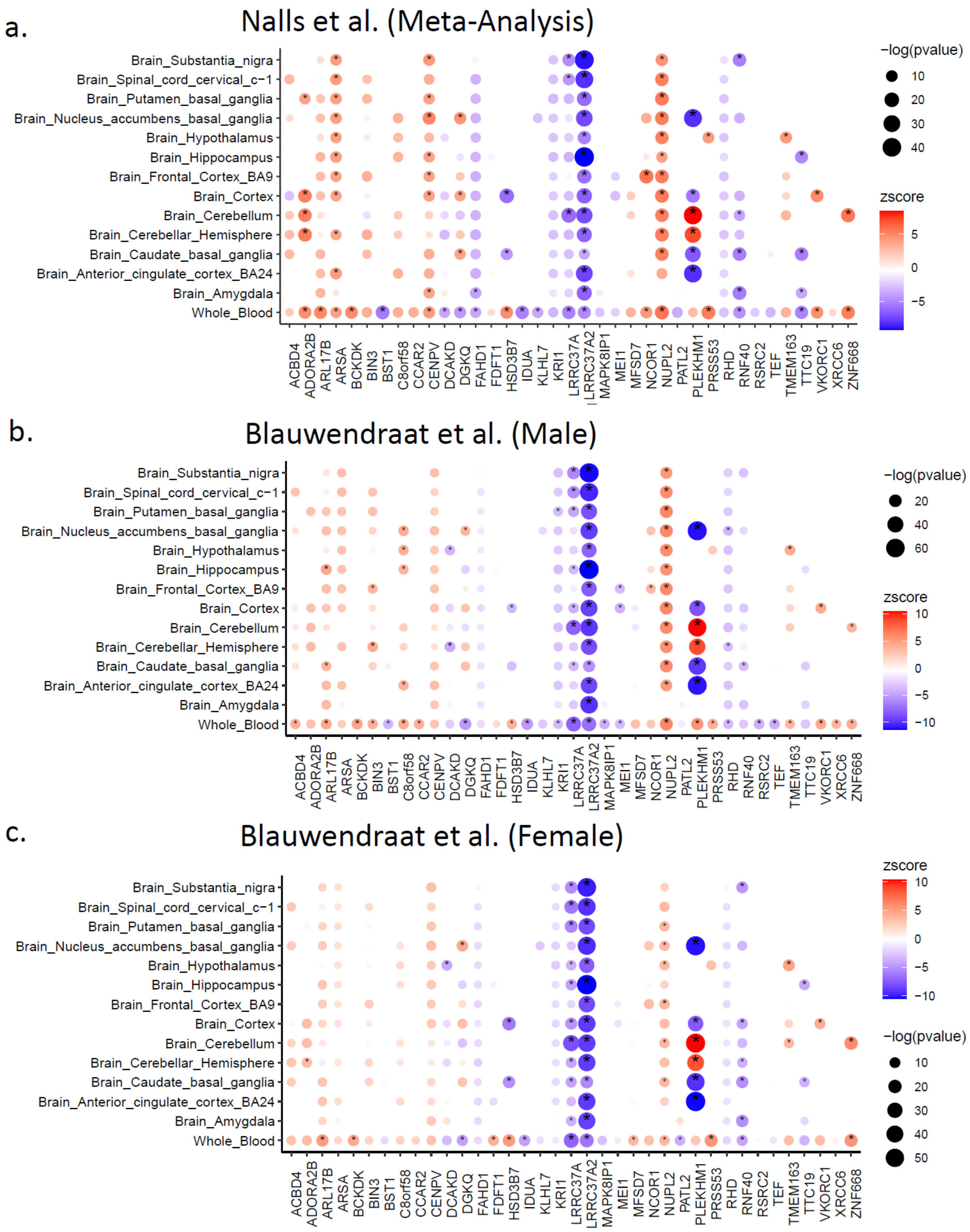

Supplementary Figure 2

Conservation of whole-blood consensus imputed gene signature (IGS) across brain tissues. Bubble plots of imputed z-scores and within-tissue Benjamini-Hochberg (BH) adjusted p-value for whole-blood IGS (37 genes) using GTEx whole-blood and brain tissue references for **a)** Blauwendraat et al. (Female), **b)** Blauwendraat et al. (Male), **c)** Nalls et al. (Meta-Analysis).

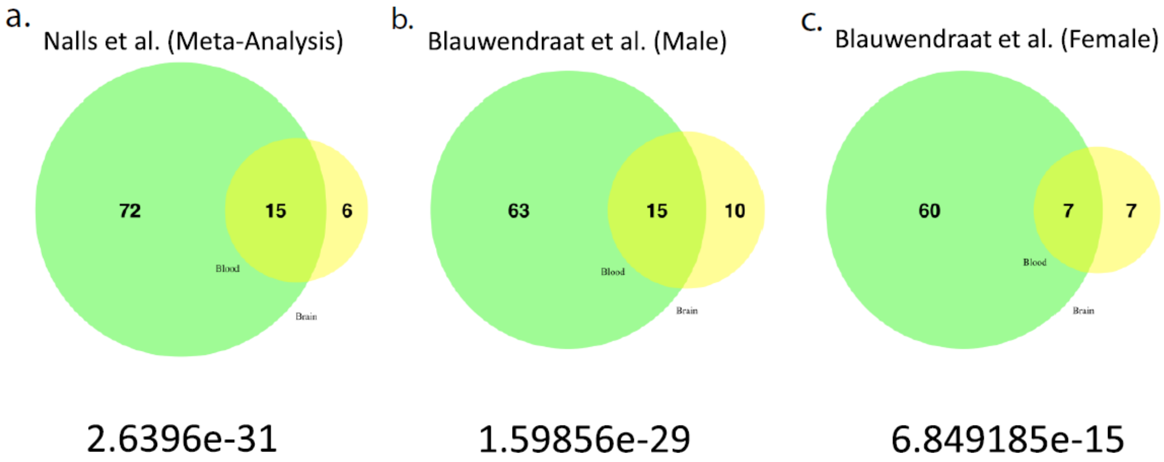

Supplementary Figure 3

Venn Diagrams of overlapping whole-blood and CNS-tissue imputed signatures for **a)** Nalls et al. (Meta-Analysis), **b)** Blauwendraat et al.(Male), **c)** Blauwendraat et al. (Female).

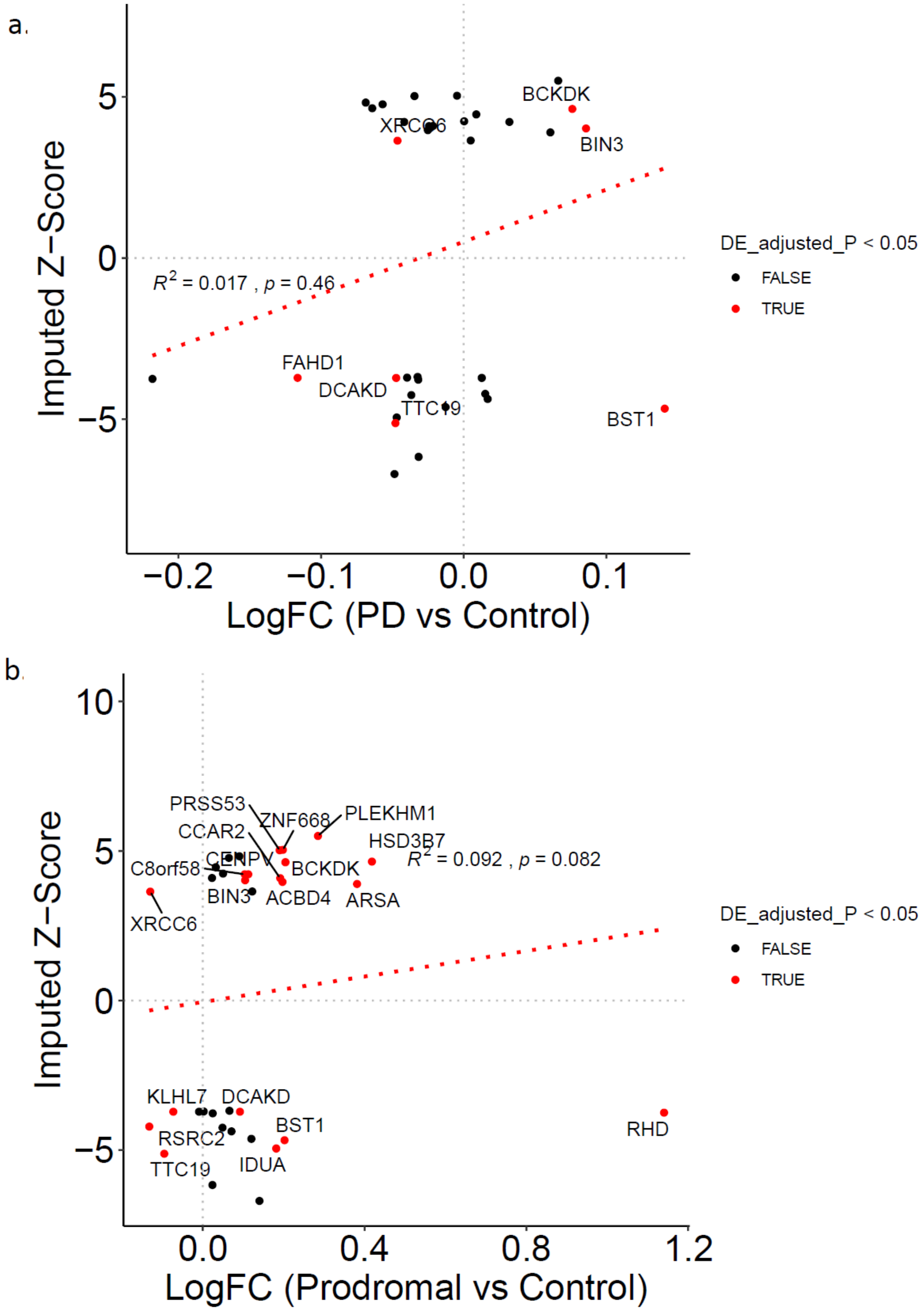

Supplementary Figure 4

Scatter plots of imputed z-scores (y-axis) and log fold changes (x-axis) for whole-blood consensus IGS (35 genes) in **a)** PD (spearman Correlation, R^2 = 0.017, p-value = 0.46) and **b)** prodromal cohorts (spearman Correlation, R^2 = 0.092, p-value = 0.082)

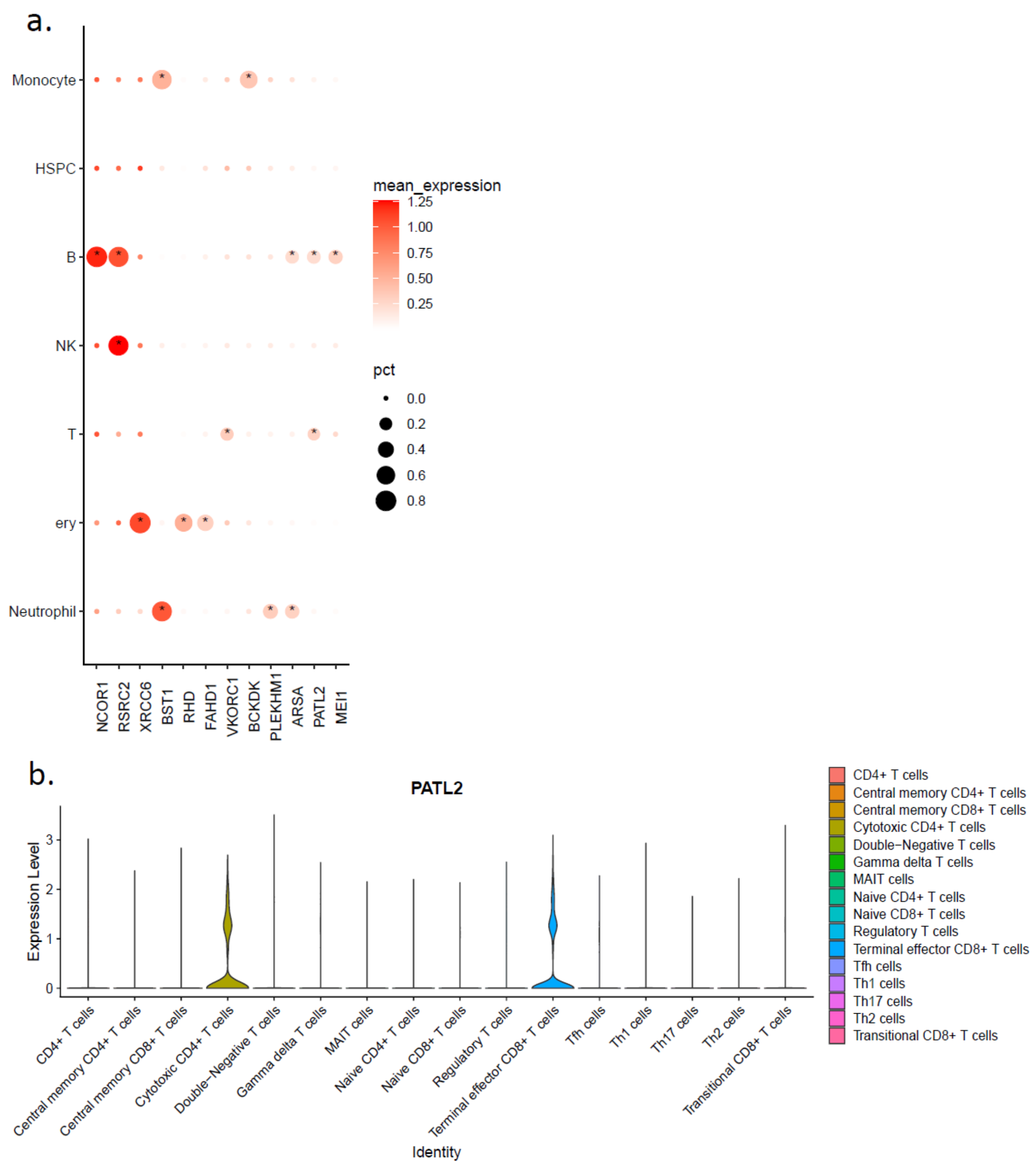

Supplementary Figure 5

a) Cell-type specific expression of whole-blood consensus IGS in single-cell RNAseq (scRNA-seq) of healthy human whole-blood (n = 7,551 cells). Only imputed risk genes with significant adjusted p-value (< 0.05) are shown. **b)** Expression of *PATL2* in T-lymphocyte subpopulations isolated from scRNA-seq of T-lymphocyte specific scRNA-seq study of whole-blood isolated from 8 PD patients and 13 healthy controls.

Supplementary Table 1
Consensus whole-blood-based imputed gene signature, derived from combining significant imputed risk genes from meta-analyses by Nalls et al. and Blauwendraat et al.

Genes	Sex	Log-fold change (males)	Log-fold change (female)	Log-fold change (shared)	Log-fold change (final)
LRRC37A	Shared	-7.79171	-7.1041	-5.19944	-6.69842
LRRC37A2	Shared	-7.29645	-6.32061	-4.88598	-6.16768
ZNF668	Shared	4.179681	5.608697	5.316974	5.035117
PRSS53	Shared	4.093328	5.571473	5.404819	5.023207
HSD3B7	Shared	3.927636	5.243719	4.764637	4.645331
ARL17B	Shared	4.400051	5.158173	4.904968	4.821064
BCKDK	Shared	4.562523	4.513018	4.795389	4.623643
IDUA	Shared	-5.18704	-4.46685	-5.18561	-4.9465
DGKQ	Shared	-5.26262	-4.37139	-4.23762	-4.62388
RNF40	Shared	0	-4.1918	-4.55495	-4.37337
FDFT1	Female	0	4.096145	0	4.096145
MFSD7	Female	0	3.827452	0	3.827452
NUPL2	Shared	6.1705	3.814253	5.637144	5.207299
PATL2	Female	0	-3.77294	0	-3.77294
PLEKHM1	Male	5.499934	0	0	5.499934
VKORC1	Shared	4.372237	0	4.537897	4.455067
TEF	Male	-4.25128	0	0	-4.25128
C8orf58	Male	4.221626	0	0	4.221626
RSRC2	Male	-4.21278	0	0	-4.21278
MAPK8IP1	Male	-4.15662	0	0	-4.15662
CCAR2	Male	4.091063	0	0	4.091063
BIN3	Male	4.020859	0	0	4.020859
ACBD4	Male	3.964453	0	0	3.964453
BST1	Shared	-3.90863	0	-5.43343	-4.67103
RHD	Male	-3.74877	0	0	-3.74877
KRI1	Male	-3.70983	0	0	-3.70983
MEI1	Male	-3.68656	0	0	-3.68656
TMEM163	Male	3.644944	0	0	3.644944
XRCC6	Male	3.638988	0	0	3.638988
TTC19	Shared	0	0	-5.12297	-5.12297
ADORA2B	Shared	0	0	4.767715	4.767715
NCOR1	Shared	0	0	4.241448	4.241448
CENPV	Shared	0	0	4.220328	4.220328
ARSA	Shared	0	0	3.896484	3.896484
DCAKD	Shared	0	0	-3.71862	-3.71862
KLHL7	Shared	0	0	-3.71818	-3.71818
FAHD1	Shared	0	0	-3.71548	-3.71548

Supplementary Table 2

Performance of GWAS-specific and consensus whole-blood-based imputed gene signature in classifying Parkinson's disease versus control, and prodromal disease versus control. Measures included accuracy, area under curve for the receiver operating curve, and area under curve for the precision recall curve.

		**PD vs Control**					
		**Male (n = 550 Cases; 550 Control)**			**Female (n = 319 Cases; 319 Control)**		
**Gene Set**	**Gene Set Size**	**Accuracy**	**AUC(ROC)**	**AUC(PR)**	**Accuracy**	**AUC(ROC)**	**AUC(PR)**
**Blauwendraat et al. (Male)**	24	61.4	64.3	64.6	64.6	68.7	70.0
**Blauwendraat et al. (Female)**	12	56.5	58.4	57.8	63.3	67.5	67.3
**Nalls et al. (Meta-analysis)**	20	60.7	63.1	64.3	66.1	69.7	71.8
**Total Imputed Gene Signature**	35	62.0	65.6	65.2	67.6	71.7	71.6
**DEGs (PD vs Control)**	35	65.0	68.4	68.4	65.8	69.3	70.6
**Average**	NA	60.2	62.8	63.0	65.4	69.4	70.2
		**Prodromal vs Control**					
		**Male (n = 198 Cases; 198 Control)**			**Female (n = 56 Cases; 56 Control)**		
**Gene Set**	**Gene Set Size**	**Accuracy**	**AUC(ROC)**	**AUC(PR)**	**Accuracy**	**AUC(ROC)**	**AUC(PR)**
**Blauwendraat et al. (Male)**	24	74.0	80.3	80.4	71.4	71.5	69.1
**Blauwendraat et al. (Female)**	12	71.2	77.0	78.0	71.4	76.0	73.9
**Nalls et al. (Meta-analysis)**	20	72.5	79.0	79.7	67.0	71.9	70.3
**Total Imputed Gene Signature**	35	74.7	82.3	83.6	73.2	75.3	73.0
**DEGs (Prodromal vs Control)**	35	73.7	80.7	81.3	70.5	76.7	77.3
**Average**	NA	73.1	79.6	80.4	70.8	73.7	71.6
